# Ageing People Living with HIV/AIDS, PLWHA; More Dental Challenges; the Romanian Dental Professional’s Point of View

**DOI:** 10.3390/life13051096

**Published:** 2023-04-27

**Authors:** Florentina Caministeanu, Loredana Sabina Cornelia Manolescu, Mădălina Adriana Malița, Viorel Ștefan Perieanu, Elena Cristina Marcov, Iuliana Babiuc, Camelia Ionescu, Cristina Florentina Pîrvu, Radu Catalin Costea, Ioana Voinescu, Liliana Burlibasa, Irina Prasacu, Mihai Burlibasa

**Affiliations:** 1 Department of Dental Technology, Faculty of Midwifery and Nursing, “Carol Davila” University of Medicine and Pharmacy, 050474 Bucharest, Romania; florentina.caministeanu@drd.umfcd.ro (F.C.); viorel.perieanu@umfcd.ro (V.Ș.P.); iuliana.babiuc@umfcd.ro (I.B.); radu-catalin.costea@umfcd.ro (R.C.C.); ioana.voinescu@umfcd.ro (I.V.); mihai.burlibasa@umfcd.ro (M.B.); 2 Department of Microbiology, Parasitology and Virology, Faculty of Midwifery and Nursing, “Carol Davila” University of Medicine and Pharmacy, 050474 Bucharest, Romania; 3 Department of Restorative Dentistry, Faculty of Dentistry, “Carol Davila” University of Medicine and Pharmacy, 0102210 Bucharest, Romania; elena.marcov@umfcd.ro; 4 Department of Dental Prostheses, Faculty of Dentistry, “Carol Davila” University of Medicine and Pharmacy, 0102210 Bucharest, Romania; camelia.ionescu@umfcd.ro; 5 Department Scientific Research Methodology—Ergonomics, Faculty of Dentistry, “Carol Davila” University of Medicine and Pharmacy, 0102210 Bucharest, Romania; cristina.pirvu@umfcd.ro; 6 Genetics Department, Faculty of Biology, University of Bucharest, 060101 Bucharest, Romania; liliana.burlibasa@bio.unibuc.ro; 7 Department of Fundamental Sciences, Faculty of Pharmacy, “Carol Davila” University of Medicine and Pharmacy, 050474 Bucharest, Romania; irina.prasacu@umfcd.ro

**Keywords:** social isolation, dental treatments, ageing people living with HIV/AIDS (PLWHA)

## Abstract

Background: In Romania, HIV (human immunodeficiency virus) and AIDS (acquired immunodeficiency syndrome) were first acknowledged in 1989. Getting older with HIV/AIDS is now possible due to antiretroviral treatment, but it can bring dental problems due to HIV itself or to the reluctance of dental professionals to treat dental problems. Our study aims to assess the attitudes, knowledge, and practices of Romanian dental professionals regarding aging PLWHA. Methods: An analytical cross-sectional observational survey based on a self-administered questionnaire was conducted for Romanian dental professionals from October 2022 to January 2023. Results: The responders’ group profile was as follows: a mean age of 39.09 ± 0.36 years (limit: 19–75), a majority of 991 (90.01%) from urban dental offices, and 364 (33.06%) with more than twenty years’ experience. A total of 517 (46.95%) responders had an unprofessional attitude and declared that, if possible, they would avoid taking part in performing dental treatments for people living with HIV/AIDS (PLWHA). There were 89 (8.08%) dental professionals that refused to work with PLWHA. Only 363 (32.97%) had worked with one previously. The dental professionals in rural areas refused PLWHA more frequently: 20% (N = 22) of rural dental professionals vs. 6.76% (N = 67) of urban dental professionals refused to work with PLWHA (OR = 0.30; 95% CI: 0.16-.56). The logistic regression applied for the 1101 responders revealed after stepwise selection that the most influential factor for their refusal to work with PLWHA in our study group was being previously exposed to HIV during dental practice (OR = 14.45; 95% CI: 8.55- 24.42; *p* = 0.000). Conclusions: Dental educators and health care planners should promote the knowledge of prophylaxis and positive attitudes towards the treatment of PLWHA. Successful resolution of these concerns is time consuming and expensive but necessary if dentists are to satisfy their professional obligations to HIV/AIDS patients.

## 1. Introduction

Romania is a country where HIV (human immunodeficiency virus) and AIDS (acquired immunodeficiency syndrome) were only first acknowledged after the fall of the communist regime in 1989 [1]. At that time, most HIV/AIDS Romanian cases (93.7%; 1094) occurred in children less than 13 years of age at diagnosis who were infected through the improper use of needles and syringes [2]. The number of Romanian HIV/AIDS-infected children represented at that time more than half of the HIV/AIDS-infected children in Europe [3,4,5]. Today, these children are adults and some of them are known as “long-term survivors”, having been born in the period of 1987–1991 [6].

In the most recent reports from the Department for Monitoring and Evaluation of HIV/AIDS in Romania, National Institute of Infectious Diseases, Dr. Matei Balș states that in Romania in 2022 there were 17,536 PLWHA, out of which 157 were in the 0–14 age category, 131 were in the 14–19 age category, and the majority, 17,248, were older than 20 years old [7]. Romanian HIV/AIDS infections are unique due to the increased percentage of HIV-1 subtype F, originating from 1983 Angolan HIV-1 strains and the long-term survivors [8]. Though in the beginning, the main route of HIV transmission in Romania was by the improper use of medical unsterilized equipment, at the moment heterosexual transmission is the principal type of HIV infection [2,4,8].

The population of HIV/AIDS-infected people is ageing all over the world, with an estimated 4 million people over the age of 50 years, a figure which has doubled since the introduction of effective antiretroviral therapy [9]. Thirty-seven years have passed since highly-effective antiretroviral therapy (ARV) was first shown to suppress HIV replication and saved millions of people’s lives all over the world. Despite this, people ageing with HIV have excess rates of morbidity and mortality, compared to those of the the general population, due to cardiovascular disease [10], malignancies [11], cognitive impairment [12], and reduced bone mineral density [13]. It is well known that HIV persists in latent reservoirs, and it is not yet known how HIV latency can be eliminated [14]. Getting older brings dental problems for almost everyone, but when speaking about people aging with HIV, things are more complicated, due to HIV itself and also to the reluctance of dental professionals to treat these patients, despite the extremely low likelihood of professional transmission [15]. In some cases, dentists play an important role and may even help in diagnosing different infections [16,17,18,19], even HIV infection. Oral hairy leukoplakia, an opportunistic infection of the Epstein Bar virus, may direct a professional’s attention to a potential HIV diagnosis [20]. There are several situations in which these health professionals may have a positive impact. Thus, it is important to assess how dental professionals feel about dental treatments in PLWHA and how they relate in their work with this category of people nowadays. In the past, several years after the HIV/AIDS pandemic had begun back in the 1990s, there was much reluctance in dental professionals toward working with and for PLWHA [21]. Despite the fact that prophylaxis is available for general infectious diseases and should be known by all healthcare workers [22,23,24,25], dental professionals may be exposed when working with PLWHA, and the risk of getting infected still exists even today. HIV pre-exposure and post-exposure prophylaxis methods are important when it comes to HIV/AIDS prevention.

Our study aims to assess the general situation of Romanian dental professionals nowadays regarding aging PLWHA after the COVID-19 pandemic, including their attitudes towards, knowledge of, and practices for this vulnerable population.

## 2. Materials and Methods

### 2.1. Study Design, Setting, and Participants

An analytical cross-sectional observational survey was conducted in Romanian dental offices over a period of three months in the fall–winter period from October 2022 to January 2023 and included all dental professionals (dentists, dental technicians, dental nurses, and dental students) that worked for the practices and those within other categories (such as as receptionists, janitor, or couriers). For an accurate design, we used “The Strengthening the Reporting of Observational Studies in Epidemiology” (STROBE) recommendations. The STROBE checklist is included in the Supplementary Materials (Supplementary Materials File S1). Being HIV-exposed during dental practice was defined as being in contact with saliva, blood, or tissue of an HIV-infected patient without being infected with HIV due to correct use of protective equipment. The goal of attendance for the present study was at least 1000 participants. The inclusion criteria for the participants were those working in a dental office for at least one year and those with Romanian citizenship. The questionnaire was able to be submitted only after answering all the questions. For the feasibility of the results, we decided to exclude dental offices for which the response rate was below 50% for the staff.

**Figure 1:**
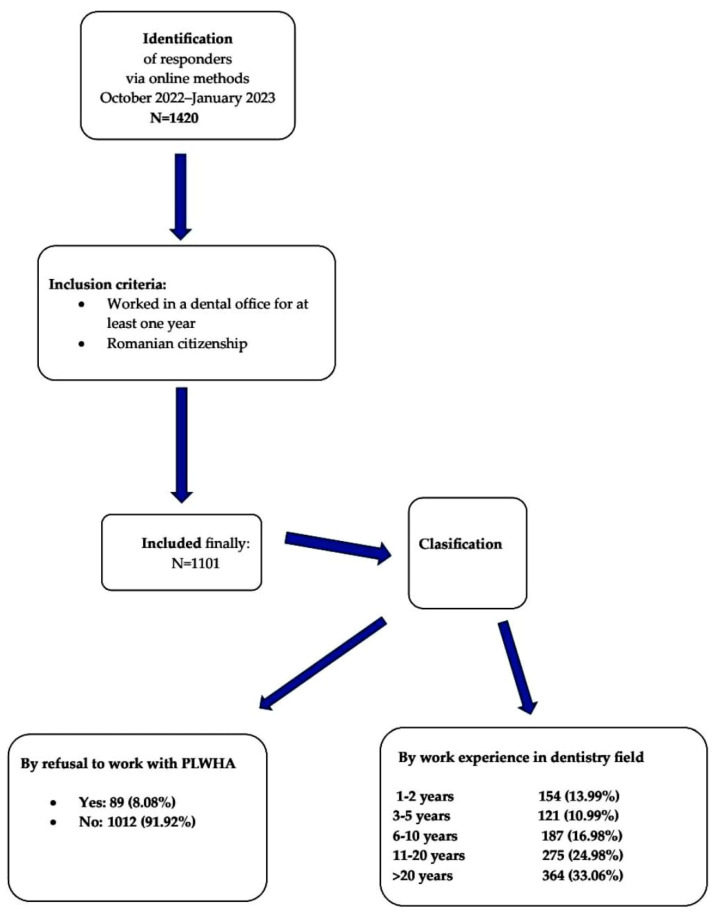
Flow diagram of the selection process.

To be sure that we had a representative sample, we used stratified sampling to preserve known groups that are important to the study. As for the sampling procedure, we applied systematic sampling. We broke down the Romanian population into the 41 counties and the capital. On the formed groups, we used simple random sampling. We selected two settlements in each county as the area of setting or residence: one town and one village. To cover urban and rural areas, we considered urban areas to be the towns, and rural areas to be the villages. Then we randomly selected one dental office in each established location. The study was conducted with the aid of medical student volunteers from the “Carol Davila” University of Medicine and Pharmacy from Bucharest, Romania. We did not select employees with certain functions in the dental offices; all employees were admitted, as shown in Figure 1.

### 2.2. Survey Questionnaire and Data Collection

A self-administered questionnaire was used to gather the data. Informed consent was requested and obtained from each responder. Participation in the online survey was voluntary and anonymous. Prior to participation, the responders were informed of the objectives of the study and each of them provided consent before responding to the questionnaire. The questionnaire was administered online using the cloud-based Google Forms survey software and was available online in October 2022, November 2022, December 2022, and January 2023. It included 26 multiple-choice questions (Supplementary Materials File S2). All questions were mandatory. The time for answering was considered adequate and it was not possible to skip questions if one did not understand the meaning of the questions.

### 2.3. Ethical Approval

The questionnaire was peer-validated and approved by the Ethics Committee of the Ploiesti Hospital, Romania (41482/9 August 2022); all the procedures in the study respected the ethical standards of the Helsinki Declaration. Informed consent was compulsory.

### 2.4. Statistical Analysis

The data from the questionnaire were analysed by means of Microsoft Excel and IBM^®^ SPSS^®^ Statistics Version 23.0 software. For data processing, the COUNTIFS function in Excel was used to filter and sort the initial database. Except for age, all of the data were categorical variables, and we applied a nonparametric Chi-square test. The variables considered significant in relation to our main dependent variable were included in a logistic regression to identify the most important predictors which contributed to the medical staff’s decision to refuse to work with PLWHA. In addition, we present the results in cross-tabulations of the entire group of respondents, as well as results from subgroups of survey respondents who either refuse to work or accept working with PLWHA. The cells of the table report the frequency counts and percentages for the number of respondents in each cell. For all tests, the threshold for statistical significance was considered to be 0.05.

## 3. Results

### 3.1. Genal Data of Responders

There were 1101 completed questionnaires. We provide thorough evidence of all the answers in File S3 of the Supplementary Materials section. The responder’s group profile was as follows: a mean age of 39.09 ± 0.36 years (limits:19–75), a majority (991; 90.01%) from urban dental offices, and 364 (33.06%) with more than twenty years’ experience in the dental field. In our group, there were 562 (51.04%) dentists, 209 (18.98%) dental technicians, 176 (15.99%) dental nurses, 66 (5.99%) dental students, and 88 (7.99%) responders working in dental offices within other categories, Table 1.

### 3.2. Attitudes towards, Knowledge about, Practice with, and Management of PLWHA

In our study group, there were 517 (46.95%) responders that declared that, if possible, they would avoid working with PLWHA. Out of the studied group, only 89 (8.08%) refused to work with PLWHA and 363 (32.97%) had worked with PLWHA. To better understand the attitudes, knowledge, practice, and management style of the dental professionals, we selected a few questions. Most of our responders knew about HIV pre- and post-exposure prophylaxis. A summary of the responders’ answers is gathered in Table 2. More than half of the dental practitioners, 616 (55.95%), were willing to get an HIV vaccine as soon as one is available.

### 3.3. More Dental Challenges and Differences in Treating Aging PLWHA, According to Area of Provenience

The majority of the dental professionals from our study, 836 (75.93%), considered ageing PLWHA to need more dental care.

We studied the differences in dental practice toward PLWHA according to the area of provenience. In the whole group of 1101 dental professionals, 991 worked in dental offices in urban areas and 110 in rural areas. We discovered that PLWHA were treated in dental offices in both urban areas and rural areas. We found no statistically significant association (*p* = 0.481) between the dental practice area and the number of PLWHA treated for dental problems: 33.29% of (N = 330) PLWHA were treated in urban dental offices vs. 30% (N = 33) of PLWHA were treated in rural dental offices. However, when we asked the dental professionals if they refused to work with PLWHA, we found a significant statistical association (*p* = 0.000) with the area of provenience of the dental office. The rural dental professionals more often refused to treat PLWHA for dental problems: 20% (N = 22) of the rural dental professionals vs. 6.76% (N = 67) of the urban dental professionals refused to work with PLWHA (OR = 0.30; 95% CI: 0.16–0.56), as shown in Table 3.

When assessing the knowledges of dental professionals according to the area of provenience of the dental office where they practiced, we discovered a statistically significant association between their knowledge of prophylaxis for general infectious diseases and the area of provenience of the dental office, *p* < 0.001. In the urban areas, there were more practitioners who knew about prophylaxis for general infectious diseases, 60.04% (N = 595), compared to those in the rural areas, 40% (N = 44). The same strong statistically significant association, *p* < 0.001, was found for their knowledge of specific pre- and post-exposure HIV prophylaxis methods. In the urban areas, 84.35% (N = 836) of the respondents knew of HIV pre-exposure prophylaxis methods and 66.59% (N = 660) knew of HIV post-exposure prophylaxis methods, compared with those in rural areas, where 70% (N = 77) of the respondents knew of HIV pre-exposure prophylaxis methods and 50% (N = 55) knew of HIV post-exposure prophylaxis methods.

In both areas of provenience, dental professionals treated PLWHA the same, irrespective of the HIV stage of infection (*p* = 0.351): 35.52% (N = 352) in the urban areas vs. 40% (N = 44) in the rural areas. The level of fear of being infected with HIV was higher in the urban dental professionals, 55.61% (N = 561), than in the rural professionals, 30% (N = 33), *p* < 0.001. Additionally, in the urban areas, the dental professionals were more willingly to get an HIV vaccine as soon as one is available (58.83%; N = 583) than the dental professionals from the rural areas (30%; N = 33), *p* < 0.001.

### 3.4. Aging with HIV, More Dental Challenges, Fear of Working with PLWHA

In our studied group of 1101 dental professional responders, when asked about whether they feared working with PLWHA, there were 429 (38.96%) dental professionals that were afraid to work with PLWHA.

We found a statistically significant association between having a fear of working with PLWHA and using enhanced protective equipment when working with them, *p* < 0.00. A total of 76.92% (N = 330) of the dental professionals were using enhanced protective equipment when working with PLWHA in the 429 group compared with 49.10% (N = 330) of the dental professionals from the 672 group who were not afraid to work with PLWHA.

Another statistically significant association with the fear of working with PLWHA was with the respondent’s knowledge of prophylaxis for infectious diseases. A total of 58.04% (N = 639) of the dental professionals in the 1101 group knew of prophylaxis for general infectious diseases. Out of them, the majority, 63.84% (N = 408) (*p* = 0.021), were not afraid to work with PLWHA, compared with 36.15% (N-231) who were afraid. This shows that knowledge of prophylaxis for infectious disease is important, as it induces more confidence while working with PLWHA.

To see which category was more aware of prophylaxis for HIV, we compared the following: category one included dentists and dental nurses who worked directly with patients and category two included dental technicians and dental students who worked indirectly with patients. The association revealed a statistically significant connection, *p* = 0.01; 63.05% of the category one (N = 507 out of 804 responders) dentists and dental nurses were more aware of HIV prophylaxis than the dental technicians and dental students of category two (51.85%, N = 154 out of 297 responders).

We noted that 46.95% (N = 517) of the responders out of the initial N = 1101 studied group would choose to avoid working with PLWHA if possible. We found a correlation with the respondents’ years of work experience in the dentistry field; the more experience our responders had, the more they would choose to avoid working with PLWHA, if possible (33.06% (N = 364) with work experience more than 20 years vs. 24.98% (N = 275) with work experience between 11 and 20 years (*p* < 0.05)).

Next, we analysed the respondents’ fear of being infected with HIV at the dental office. A total of 53.95% (N = 594) of the 1101 group of dental professionals were afraid to get infected with HIV working at the dental office. We discovered that there is a strong association of this fear with the refusal to work with PLWHA, *p* < 0.001; 13.13% (N = 78) of responders refused to work with PLWHA due to the fear of getting infected with HIV while working at the dental office vs. 2.21% (N = 11) of responders refused to work with PLWHA for other reasons.

The logistic regression applied on the 1101 responders revealed after the stepwise selection that the most influential factor for the refusal to work with PLWHA in our study group was being previously exposed to HIV during dental practice (OR = 14.45; 95% CI: 8.55–24.42), *p* = 0.000. We calculated the odds ratio (OR) and confidence interval at the 95% level (CI), Table 3. The other influential factor for the refusal to work with PLWHA was the area of residence.

## 4. Discussion

HIV/AIDS patients or PLWHA are a special category in dental practice. The introduction of HAART (highly active anti-retroviral therapy) prolonged patients’ lives and lessened their side effects [6,26,27,28], but dental problems are still affecting their lives and wellbeing.

Oral health problems can significantly alter PLWHA’s quality of life, so dental care is necessary. Dental problems of aging PLWHA are due to several reasons. First, there are opportunistic infections with localization in the oral cavity [29]. Second, there are many barriers and less facilitators for dental care in aging PLWHA, such as dentists’ reluctance to treat them, and as a result they have more unmet needs for dental care [30].

There are many studies all over the world regarding HIV and dental practice. The danger of getting infected with HIV while working with PLWHA has been discussed since the discovery of HIV/AIDS, since HIV can be detected in all body fluids, such as saliva, blood, tears, and others [31]. Becoming infected with HIV while practicing dentistry may impact your future as well, since there is a greater possibility of transmitting HIV to one’s children, which results in another stressful situation [32].

One important factor that we came across in many studies was the discrepancy in providing dental treatments to PLWHA, due to various reasons. A study from Nepal used a mixed-effects logistic regression to predict the likelihood of dental treatments for uninfected HIV patients and PLWHA. Overall dental professionals felt uncomfortable providing dental treatments to PLWHA, with only 29% of them comfortable with performing prophylaxes and fillings, 18% with orthodontic treatments, 16% with endodontic treatments, 12% with periodontal scaling, and only 10% with extractions [33].

In a study from Brazil, to compare the knowledge, presence, and manifestations of discriminatory and stigmatizing acts of dental professionals on PLWHA, a z-test for proportions (*p* ≤ 0.05) was used. The three groups compared were dental surgeons (N = 219) and dental nurses (N = 152) in 40 municipalities and dental students at a public university (N = 179) [34]. The conclusion of the study was that dental nurses and students had low general knowledge about infectious diseases and discriminatory, stigmatizing attitudes toward PLWHA when compared with the dental surgeons. Regarding infection, the fear of contracting HIV/AIDS was more representative; a higher proportion of assistants (47.4%) believed conduct should differ in the care of PLWHA [34].

PLWHA have several complaints when it comes to dental treatments, such as delayed access to dental services and factors related with delayed access to dental services. A cross-sectional study using a self-report questionnaire completed by 299 PLWHA conducted at the largest HIV research clinic in Thailand during 2009–2010 revealed that the majority, 84.3%, reported having several dental conditions and wished to have access to specialist services, if possible. Because they have been discriminated against by dental staff, the majority, 242 subjects (80.9%) would not disclose their HIV status when seeing a dentist, even if they trusted their dentists to act morally. The most cited reason of such behavior was being afraid of not receiving dental treatment from the dentists or staff (51.7 and 40.9%, respectively). The PLWHA responders had good basic knowledge of oral health with regard to HIV infection and experienced common dental problems [35].

The same situation was shown in a study from Iran in 2019 [36], which found that although the life expectancy of PLWHA has increased, oral manifestations can affect the oral-health-related quality of life (OHRQoL) of these patients. PLWHA were worried about their dental health problems. The OHRQoL was significantly better in denture-wearing patients. In this cross-sectional study, 250 PLWHA were randomly selected from the Shiraz Voluntary Counseling and Testing Center. Their OHRQoL was measured using the revised Geriatric Oral Health Assessment Index. The mean score of the patients’ OHRQoL was 24.55 ± 6.27. The lowest and highest scores belonged to the psychosocial and pain categories, respectively. In a univariate analysis, the OHRQoL was significantly associated with the patient’s age (*p* = 0.012), duration of the disease (*p* = 0.009), job (*p* = 0.006), edentulous status (*p* = 0.003), and whether or not they wore dentures (*p* < 0.001). However, in a multiple linear regression analysis, a significant difference was found only between denture-wearing and non-denture-wearing patients (*p* ≤ 0.001) [36].

Most of PLWHA’s complaints regarding dental treatment come from the refusal or fear of dental professionals to treat them, including the way PLWHA are viewed by dental professionals. Older studies from 1987 have discussed the significance of HIV/AIDS in the practice of dentistry, revealing the perceived stigma of treating such patients, together with the fear that HIV is transmitted through dental treatment and the reluctance of the dental professionals to care for PLWHA [37].

There are Romanian studies that discuss health-related quality of life (HRQoL) in people living with HIV (PLWHA). Their conclusions were that anxiety/depression symptoms are frequently reported by PLHIV in Romania: 50% (from a study group of 570 PLHIV) vs. 30% (of the Romanian population) [38]. In a multivariable logistic regression, there were two factors associated with worse HRQoL in PLHIV: having a bad or very bad self-rated health status and the presence of a mental health condition. This study also revealed that in Romania, being gay/bisexual and being disabled/unemployed were associated with worse HRQoL [38]. Unfortunately, few studies have discussed the issue of HIV/AIDS in the context of dental practice in Romania, where there are approximatively 17,536 people living with HIV/AIDS [7]. In fact, according to our knowledge, this is the first study that has conducted an assessment of the attitudes, knowledge, and practices of Romanian dental professionals regarding HIV-infected aging patients.

We conducted an analytical cross-sectional observational survey based on a self-administered questionnaire in Romanian dental offices to study all dental professionals involved in the dental care of HIV/AID patients from October 2022 to January 2023. The responders’ group profile revealed a median age of forty years with the range from nineteen to seventy-five years. The majority of the dental professionals, 991 (90.01%), worked in urban dental offices. A dentist’s attitude towards patients should meet professional expectations. In our study group, almost half, 517 (46.95%), declared that, if possible, they would avoid working with PLWHA, which is considered a very unprofessional attitude. In our study, we found more dental professionals with this attitude when compared with the number of dental professionals from an Indian study.

In particular, the Indian study involved 206 dentists practicing in the Trichur district of Kerala and aimed to reveal discrimination by some healthcare workers, including dentists, towards PLWHA. Out of 206 participants, 39.3% were unwilling to treat HIV patients. Senior dentists showed more reluctance to treat PLWHA [39], similar with our findings. In our study, we found a correlation with the participants’ work experience in the dentistry field. The more experience our responders had, the more likely they would choose to avoid working with PLWHA, if possible: 33.06% (N = 364) with more than 20 years’ work experience vs. 24.98% (N = 11–20 years, *p* < 0.05).

A literature review of forty-nine studies from 2001 to 2016 addressing occupational health problems among dental practitioners revealed that percutaneous injuries especially among young dentists and students were a concern, a reason for the fear of becoming infected with HIV/AIDS during dental practice [40]. In our study, the logistic regression applied on the 1101 responders revealed that the most influential factor for the refusal to work with the HIV-infected patients was being previously exposed to HIV during dental practice (OR = 14.45; 95% CI: 8.55–24.42), *p* = 0.000. There were 89 (8.08%) dental professionals that refused to work with PLWHA in our 1101 study group. The rural dental professionals refused to treat PLWHA more often: 20% (N = 22) of the rural dental professionals vs. 6.76% (N = 67) of the urban dental professionals refused to work with PLWHA (OR = 0.30; 95% CI: 0.16–0.56).

Recent studies of HIV-related healthcare stigma/discrimination and unmet needs among people living with HIV (PLHIV) in England and Wales from 2021 have shown that the problem still exists. A cross-sectional probability survey of 3475 PLHIV provided complete data. Modified Poisson regression models with log links and robust variance estimators were used to produce prevalence ratios and 95% confidence intervals for unadjusted and adjusted associations between patients’ demographic characteristics, HIV-related healthcare stigma/discrimination (individual items and total scale score), and unmet needs variables. Two in five participants (40%) agreed with at least one HIV-related healthcare stigma/discrimination item [41].

It is essential for dental professionals to have adequate knowledge of prophylaxis for general infectious diseases [42,43,44], HIV/AIDS prophylaxis, and the implications in dental practice when working with PLWHA. In our Romanian study, there were 363 (32.97%) dental professionals who had worked with at least one HIV-infected person. Assessing the knowledge of the dental professionals was an important issue. Prophylaxis for general infectious diseases was known by more than half of the responders, 639 (58.04%), while the majority, 913 (82.92%), knew of HIV pre-exposure prophylaxis methods. Fewer dental professionals, only 715 (64.94%), knew of HIV post-exposure prophylaxis methods. Our findings were higher when compared to those of a recent study from Saudi Arabia in 2022. Participants in this study showed a low knowledge of safety regarding HIV (39.5%) and almost 50% of the responders expressed an unwillingness to treat HIV-positive patients, as well as inadequate knowledge about and an unprofessional attitude towards PLWHA [45].

### Limitations of the Study

This study was subject to several potential limitations. The study was limited by its cross-sectional design. The representativeness of the study sample is limited due to the default selection of respondents who have digital skills, as the studied sample mainly analyzed people who could use the internet and electronic documents. Another limitation of this study was that it analyzed only a short period of time of 4 months and included a limited number of participants (1101). Our study is important since no other recent study has covered PLWHA dental care in Romania. It may reveal to the medical community the real situation of PLWHA in Romanian dental offices and draw attention to potential changes; therefore, monitoring this issue may be useful.

## 5. Conclusions

Dental coverage for PLWHA is essential, so that they can have timely access to dental care. Many of the barriers to work with PLWHA can be addressed and overcome by management services. In Romania, there are dental professionals who refuse to work with PLWHA, the main reason being having been previously exposed to HIV during dental practice. Having this in mind, we conclude that education is beneficial when working with HIV-infected patients, and more educational programs regarding prophylaxis for general infectious diseases and HIV pre- and post-Exposure prophylaxis methods should be in place in dental offices in training programs for all dental professionals. Dental educators and healthcare planners should promote knowledge about prophylaxes and positive attitudes towards the treatment of PLWHA. The successful resolution of these concerns is time consuming and expensive but necessary if dentists are to satisfy their professional obligations to PLWHA.

## Figures and Tables

**Table 1:** General socio-demographic characteristics of respondents to the questionnaire. There was a total of 1101 responders: 1012 responders that did not refuse to work with PLWHA and 89 responders that refused to work with PLWHA.

Characteristic	Total Asked = 1101	Total Asked = 1012	Total Asked = 89
age, median (min–max), years	40 (19–75)	40 (19–75)	40(28–50)
rural residence (N)	110	177	22
P%	9.9%	17.49%	24.72%
work experience in dentistry field			
1–2 years, (N)	154	154	0
P%	13.99%	15.22%	
3–5 years, (N)	121	121	0
P%	10.99%	11.96%	
6–10 years, (N)	187	154	33
P%	16.98%	15.22%	37.08
11–20 years, (N)	275	242	33
P%	24.98%	23.91%	37.08
>20 years, (N)	364	341	23
P%	33.06%	33.70%	25.84
have worked with PLWHA, (N)	363	319	67
P%	32.97%	31.52%	75.28
knew of prophylaxis for general infectious diseases, (N)	639	583	56
P%	58.04%	57.60%	62.92%

**Table 2:** Attitudes towards, knowledge about, and management of PLWHA. There were 1101 total responders: 1012 responders that did not refuse to work with PLWHA and 89 responders that refused to work with PLWHA.

	Total Asked = 1101	Total Asked = 1012	Total Asked = 89	*p* Value
HIV Attitude, knowledge, management	yes	yes	yes	
know of HIV pre-exposure prophylaxis methods, (N)	913	858	55	<0.05
P%	82.92%	84.78%	61.79%	
know of HIV post-exposure prophylaxis methods, (N)	715	660	55	>0.05
P%	64.94%	65.21%	61.79%	
have been exposed to HIV during dental practice, (N)	254	187	67	<0.05
P%	23.07%	18.47%	75.28%	
treat PLWHA differently according to HIV treatment or stage of disease, (N)	396	352	44	>0.05
P%	35.97%	34.78%	49.43%	
are afraid to get infected with HIV in the dental office, (N)	594	528	66	<0.05
P%	53.95%	52.17%	74.15%	
have taken ARV for HIV post-exposure prophylaxis, (N)	33	11	22	<0.05
P%	3%	1.08%	24.71%	
are using enhanced protective equipment when working with PLWHA, (N)	660	583	77	<0.05
P%	59.95%	57.60%	86.51%	
consider ageing PLWHA to need more dental care, (N)	836	770	66	>0.05
P%	75.93%	76.08%	74.15%	
are afraid to work with PLWHA, (N)	429	363	66	<0.05
P%	38.96%	35.86%	74.15%	
refuse to speak with PLWHA, (N)	66	33	33	<0.05
P%	5.99%	3.26%	37.07%	

**Table 3:** Logistic regression for the 1101 people in the total studied group who refused to work with PLWHA.

	B	E.S.	Wald	*p*	OR	Lower CI 95%	Upper CI 95%
area of residence of dental practice (urban vs. rural)	−1.181	0.308	14.735	0.000	0.307	0.168	0.561
know of HIV post-exposure prophylaxis methods	−0.454	0.257	3.112	0.078	0.635	0.384	1.052
previously exposed to HIV during dental practice	2.671	0.268	99.698	0.000	14.456	8.557	24.420

Abbreviations: B (coefficient), E.S. (standard error), Wald (Wald test), *p* (*p*-value), OR (odds ratio), CI (confidence interval).

## Supplementary Materials

The following supporting information can be downloaded at: https://www.mdpi.com/article/10.3390/life13051096/s1, File S1: The STROBE checklist; File S2: The administered questionnaire; File S3: The questionnaire responses.

## Abbreviations

AIDS	acquired immunodeficiency syndrome
ARV	antiretroviral therapy
HAART	highly active anti-retroviral therapy
HIV	human immunodeficiency virus
HRQoL	health-related quality of life
OHRQoL	oral-health-related quality of life
PLHIV	people living with HIV
PLWHA	people living with HIV/AIDS
