# Supplementary material for: Ageing People Living with HIV/AIDS, PLWHA; More Dental Challenges; the Romanian Dental Professional’s Point of View
Source: Life (Basel). 2023 Apr 27;13(5):1096. doi: 10.3390/life13051096 (PMC10224099; doi:10.3390/life13051096)
Supplement: Supplementary file 1 [file life-13-01096-s001.zip › File S2 The administered questionnaire.pdf]

Ageing People Living with HIV; more dental challenges

STUDY ON THE ROMANIAN DENTAL PROFESSIONAL'S POINT OF VIEW

INFORMED CONSENT

QUESTIONNAIRE ADDRESSED TO PERSONS OVER 18 YEARS OLD

Hello!

We invite you to contribute to our research regarding dental challenges that ageing people with HIV must face in Romania. We are assessing fears, beliefs, realities, attitudes, knowledge, practice and management. The questionnaire to be completed has several sections and is addressed to all dental professional that work in the dental office: dentists, dental technicians, dental nurses, dental students that work for practice and other categories (such as receptionist, janitor, and curries). The completion time is approximately 6 minutes. Participation is voluntary and your answers will be treated confidentially, the information you provide will only be used for research purposes. You have the freedom at any time to withdraw from the study, to interrupt or to resume the questionnaire. The research complies with the international ethical recommendations regarding the absolute confidentiality of the data collected in the study as well as the anonymity and safety of the respondents' data. The requirements of EU Regulation 2016/79, regarding the protection of natural persons regarding the processing of personal data and the free movement of such data and of Law no. 506/2004, regarding the processing of personal data and the protection of private life in the electronic communications sector. It is important that during the study you answer the questions as honestly as possible in order to draw correct conclusions.

For any clarification related to this study, during or after its completion, you can contact us by email at loredana.manolescu@umfcd.ro

QUESTIONS

1. What is your role in the dental practice? dentists, dental technicians, dental nurses, dental students, other (such as receptionist, janitor, and curries.
2. How old are you? ________
3. How long have you been practicing in the dental office? _______
4. Do you know infectious diseases prophylaxis, were you trained for this? Yes/no
5. Where is the dental office where you practice located? rural/urban
6. You are vaccinated against: VHA/VHB/influenza/COVID-19/tetanus
7. Have you worked with HIV infected patients? Yes/no
8. Do you know HIV Pre-Exposure Prophylaxis methods? Yes/no
9. Do you know HIV Post- Exposure Prophylaxis methods? Yes/no
10. Have been HIV exposed during dental practice? Yes/no
11. Did you refuse to work with an HIV infected patient? Yes/no
12. If you can choose, would you chose to avoid treating an HIV infected patient? Yes/no

13. How do you find out that your patient is HIV infected? I ask directly all the patients/I run a questionnaire/I do not ask
14. Are you afraid to get HIV infected working in the dental office? Yes/no
15. If the patient is HIV infected, you: are more careful than usual/ refuse to treat the patient/do nothing special
16. Have you taken ARV for HIV Post- Exposure Prophylaxis? Yes/no
17. When you work with an HIV infected patient do you treat them different according to HIV stage or treatment? Yes/no
18. Are you using enhanced protective equipment when working with HIV infected? Yes/no
19. HIV is transmitted: by direct contact or touch/through body fluids, blood/sexual/mother to child at birth
20. HIV is diagnosed by: rapid tests/ rT-PCR/serology tests
21. Have you worked with an HIV infected patient? Yes/no
22. Do you consider that ageing HIV patients need more dental care? Yes/no
23. Are you afraid to work with an HIV infected patient? Yes/no
24. Did you refuse to speak with an HIV infected patient in the dental office? Yes/no
25. Are you HIV infected? Yes/no
26. Are you willing to get an HIV vaccine as soon as one will be available.? Yes/no

THANK YOU!
