# Supplementary material for: Ageing People Living with HIV/AIDS, PLWHA; More Dental Challenges; the Romanian Dental Professional’s Point of View
Source: Life (Basel). 2023 Apr 27;13(5):1096. doi: 10.3390/life13051096 (PMC10224099; doi:10.3390/life13051096)
Supplement: Supplementary file 1 [file life-13-01096-s001.zip › File S3 The questionnaire responses.pdf]

Responses to the questionnaire

1. What is your role in the dental practice? dentists, dental technicians, dental nurses, dental students, other (such as receptionist, janitor, and curries).

dentists	562	51.04%
dental technicians	209	18.98%
other	88	7.99%
dental nurses	176	15.99%
dental student	66	5.99%

2. How old are you?

Mean	39.09991
Standard Error	0.363241
Median	40

3. How long have you been practicing in the dental office?

1-2 years	154 (13.99%)
3-5 years	121 (10.99%)
6-10 years	187 (16.98%)
11-20 years	275 (24.98%)
>20 years	364 (33.06%)

4. Do you know infectious diseases prophylaxis, were you trained for this? Yes/no 369 (58.04%)/ 462 (41.96%)
5. Where is the dental office where you practice located? rural/urban
   110 (9.9%)/991 (90.01%)
6. You are vaccinated against: VHA/VHB/influenza/COVID-19/tetanus
   112/987/569/789/1101
7. Have you worked with HIV infected patients? Yes/no

		P%
yes	386	35.06
no	715	64.94

8. Do you know HIV Pre-Exposure Prophylaxis methods? Yes/no

		P%
yes	913	82.92
no	188	17.08

9. Do you know HIV Post- Exposure Prophylaxis methods? Yes/no

		P%
yes	715	64.94
no	386	35.06

10. Have been HIV exposed during dental practice? Yes/no

		P%
yes	254	23.07
no	847	76.93

11. Did you refuse to work with an HIV infected patient? Yes/no

		P%
yes	89	8.08
no	1012	91.92

12. If you can choose, would you choose to avoid treating an HIV infected patient? Yes/no

		P%
yes	517	46.96
no	584	53.04

13. How do you find out that your patient is HIV infected? I ask directly all the patients/I run a questionnaire/I do not ask

396/618/87

14. Are you afraid to get HIV infected working in the dental office? Yes/no

		P%
da	594	53.95
nu	507	46.05

15. If the patient is HIV infected, you are more careful than usual/ refuse to treat the patient/do nothing special

		P%
do nothing special	396	35.97
refuse to treat the patient	77	6.99
you are more careful than usual	628	57.04

16. Have you taken ARV for HIV Post- Exposure Prophylaxis? Yes/no

		P%
yes	33	3.00
no	1068	97.00

17. When you work with an HIV infected patient do you treat them different according to HIV stage or treatment? Yes/no

		P%
yes	396	35.97
no	705	64.03

18. Are you using enhanced protective equipment when working with HIV infected? Yes/no

		P%
yes	660	59.95
no	441	40.05

19. HIV is transmitted: by direct contact or touch/through body fluids, blood/sexual/mother to child at birth

66/1101/900/809

20. HIV is diagnosed by: rapid tests/ rT-PCR/serology tests

37/1101/667

21. Have you worked with an HIV infected patient? Yes/no

		P%
yes	363	32.97
no	738	67.03

22. Do you consider that ageing HIV patients need more dental care? Yes/no

		P%
yes	836	75.93
no	265	24.07

23. Are you afraid to work with an HIV infected patient? Yes/no

		P%
yes	429	38.96
no	672	61.04

24. Did you refuse to speak with an HIV infected patient in the dental office? Yes/no

		P%
yes	66	5.99
no	1035	94.01

25. Are you HIV infected? Yes/no

0/1101

26. Are you willing to get an HIV vaccine as soon as one will be available.? Yes/no

		P%
yes	616	55.95
no	485	44.05
